# Influence of the Print Envelope Temperature on the Morphology and Tensile Properties of Thermoplastic Polyolefins Fabricated by Material Extrusion and Material Jetting Additive Manufacturing

**DOI:** 10.3390/polym15183785

**Published:** 2023-09-16

**Authors:** Lukas Hentschel, Sandra Petersmann, Frank Kynast, Ute Schäfer, Clemens Holzer, Joamin Gonzalez-Gutierrez

**Affiliations:** 1 Polymer Processing, Montanuniversitaet Leoben, 8700 Leoben, Austria; lukas.hentschel@stud.unileoben.ac.at (L.H.); clemens.holzer@unileoben.ac.at (C.H.); 2 Materials Science and Testing of Polymers, Montanuniversitaet Leoben, 8700 Leoben, Austria; s.petersmann@cuas.at; 3 ARBURG GmbH + Co. KG, 72290 Lossburg, Germany; 4 Research Unit Experimental Neurotraumatology, Department of Neurosurgery, Medical University of Graz, 8036 Graz, Austria; ute.schaefer@medunigraz.at; 5 BioTechMed-Graz, 8010 Graz, Austria

**Keywords:** additive manufacturing, ARBURG plastic freeforming, material extrusion, polyolefin, morphology, crystallinity, mechanical properties, infill orientation

## Abstract

Additive manufacturing (AM) nowadays has become a supportive method of traditional manufacturing. In particular, the medical and healthcare industry can profit from these developments in terms of personalized design and batches ranging from one to five specimens overall. In terms of polymers, polyolefins are always an interesting topic due to their low prices, inert chemistry, and crystalline structure resulting in preferable mechanical properties. Their semi-crystalline nature has some advantages but are challenging for AM due to their shrinkage and warping, resulting in geometrical inaccuracies or even layer detaching during the process. To tackle these issues, process parameter optimization is vital, with one important parameter to be studied more in detail, the print envelope temperature. It is well known that higher print envelope temperatures lead to better layer adhesion overall, but this investigation focuses on the mechanical properties and resulting morphology of a semi-crystalline thermoplastic polyolefin. Further, two different AM technologies, namely material jetting (ARBURG plastic freeforming—APF) and filament-based material extrusion, were studied and compared in detail. It was shown that higher print envelope temperatures lead to more isotropic behavior based on an evenly distributed morphology but results in geometrical inaccuracies since the material is kept in a molten state during printing. This phenomenon especially could be seen in the stress and strain values at break at high elongations. Furthermore, a different crystal structure can be achieved by setting a specific temperature and printing time, also resulting in peak values of certain mechanical properties. In comparison, better results could be archived by the APF technology in terms of mechanical properties and homogeneous morphology. Nevertheless, real isotropic part behavior could not be managed which was shown by the specimen printed vertically. Hence, a sweet spot between geometrical and mechanical properties still has to be found.

## 1. Introduction

Additive manufacturing (AM) has become an important manufacturing technology over the last decades due to its high freedom of design and rapid production. Especially in medicine and healthcare, more and more beneficial use cases are found every day [1,2,3,4]. Furthermore, the variety of materials for such applications has significantly increased since new materials are being developed and validated consistently for 3D printing, provided in different shapes such as granules, filaments, viscous resins, or powders suiting the different AM methods [5,6,7]. Materials used for medical applications must fulfill several criteria depending on the category of the applications according to the Food and Drug Administration (FDA) or Medical Device Directive (MDD). Since every production step needs to be certified, the medical certification of granules may be easier since they have to undergo fewer production steps compared to filaments or powders [8].

**Figure 1:**
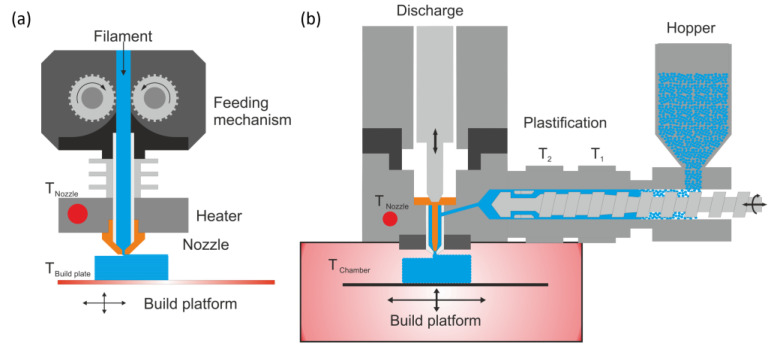
Schematic illustration of the (**a**) filament-based MEX and the (**b**) ARBURG plastic freeforming technologies.

More limitations on the material come from the AM method and the machine itself. Material extrusion (MEX) technologies are the most used methods, mainly using filaments as feedstock. Such machines can range from simple desktop printers to large industrial machines, with more features and more accurate control. The basic technology remains the same for filament-based MEX. A feedstock filament is pulled and pushed by a conveying unit, usually consisting of two grooved wheels. Further, the solid filament is fed through the cold end to the hot end, where the material is melted. The cold end is responsible for cooling the area and the filament down to prevent premature liquefaction of the polymer since the filament must still act as a piston to push the molten polymer through the nozzle. The most common diameter used for the nozzle is 0.4 mm; hence, a small strand of the material is then extruded and deposited on a moving platform relative to the nozzle. The tool head is the combination of the conveying unit, hot and cold end, as well as nozzle. A schematic display of this method is given in Figure 1a. Essential properties of the filament polymer are stiffness to provide the needed extrusion pressure, relatively low viscosity to reduce the extrusion resistance, high heat transfer potential for a uniform temperature distribution, and good welding behavior to provide a reasonable inter- and intralayer strength [9,10,11].

However, other MEX methods and AM technologies, such as the ARBURG plastic freeforming (APF) technology, can process granules instead of filaments. The corresponding machine, the freeformer, uses an injection molding unit for melting the granules and applying the needed processing pressure on the melt. A piezo-electronic shut-off valve, opening and closing up to 250 times per second, is then used to form polymeric melt droplets. Hence, the extrusion is discontinuous as one of the significant differences between the APF and the previously described MEX technology. Although droplets are theoretically formed, an interconnected chain of droplets is produced and deposited on a moving build platform [8,12]. The part formation occurs in a heated chamber and hence in a controlled environment, as a further difference to most MEX machines. Consequently, a controlled print envelope temperature can strongly influence material properties, especially for semi-crystalline polymers [12,13,14,15]. A schematic display is given in Figure 1b. Due to the droplet formation, this method is also referred to as material jetting technology, according to DIN EN ISO/ASTM 52900 [16].

These technologies show different advantages and disadvantages and clearly have different use cases. The MEX method, with a rather easy setup, can provide a rather inexpensive solution for the fabrication of 3D objects; however, it might lack accuracy due to machine or filament deviations, as well as filament slippage. Print jobs can even fail due to filament grinding or buckling, especially when dealing with elastomeric thermoplastics. These problems are not likely to occur in the APF process since granules are molten and due to the shut-off nozzle, a controlled extrusion volume is possible. However, the APF is limited in extrusion volume, because of the nozzle size, droplet volume, and operation frequencies, whereas the MEX can be applied with nozzle diameters up to 1.2 mm, and hence, can build big parts rather fast [11]. Nevertheless, Table 1 shows some key parameters of the different technologies as an overview.

Semi-crystalline polymers like polypropylene (PP) are challenging to process using AM technologies due to their crystallization kinetics and accompanying shrinkage and warpage phenomena. However, semi-crystalline polymers show desirable properties for many applications. Hence, some unique grades have been developed to improve their processability [13,17,18,19]. For semi-crystalline polymers, a higher degree of crystallinity and larger crystals generally yield a preferable s [20]. Specifically, for generative manufacturing layer-by-layer, weak bonding between individual layers leads to less mechanical performance or even layer detachments [21]. Petersmann et al. [22] showed that increasing the melt temperature of the extrudate and decreasing the printing speed resulted in a more preferable crystalline structure of PP. Ovlaque et al. [23] further studied the temperature evaluation at defined positions during printing at the APF method and analyzed the crystallinity due to the thermal history. It was found that even in a single print, the thermal history in different locations of a printed part can change and lead to different morphology, internal stresses warping, and hence, weak spots of the manufactured parts. It was proven that a bead is remelted several times during the printing cycle. The higher thermal energy input enhances interlayer bonding and mechanical performance. A higher thermal energy input can also be achieved by evaluating ambient temperatures, influencing the cooling behavior and, therefore, crystal growth. 

Further physical phenomena influencing bonding and mechanical properties are the diffusion and coalescence recently studied by Benié et al. [24]. Diffusion can also be of high interest for semi-crystalline polymers since diffusion is suppressed after forming crystals. Hence, a high cooling rate and, therefore, freezing of the polymer melt lead to a low diffusion and low bonding for semi-crystalline polymers. Further, coalescence is highly linked to diffusion and leads to a reduction in voids. It describes the formation of a single cylinder out of two individual cylinders over time and can be characterized by neck growth in the bonding area. This phenomenon is also temperature-dependent and is reasonably faster compared to diffusion [25,26].

As aforementioned, warping is one of the major issues with 3D printing semi-crystalline polymers. Warping occurs due to anisotropic shrinkage due to temperature inhomogeneities present in the AM process [27,28]. This effect is further amplified by the high shrinkage potential caused by a large volume contraction during crystallization. Hence, shrinkage and warpage behavior can only be improved by changing the polymer morphology, such as the molecular weight, polydispersity, and tacticity, or blending with a rigid filler [29,30]. Furthermore, by incorporating comonomers such as ethylene, highly elastic thermoplastic materials are obtained, and reduced shrinkage can be observed. These polymers are referred to as thermoplastic polyolefins (TPOs) and belong to the thermoplastic elastomer (TPE) group due to their high ductility and low stiffness. Such materials are particularly interesting in numerous applications attributable to their rubber-like properties and thermoplastic processability [18,31,32]. However, incorporating ethylene into a polypropylene matrix reduces the polymer’s crystallinity, which can improve shrinkage but result in a complex tensile behavior, especially at high elongations [33].

Some studies have already shown the processability of TPEs utilizing filament-based MEX AM, but this use is limited to a specific stiffness/hardness since the needed pressure for the extrusion must be transmitted via the filament. Hence, print processes might suffer from unreliable extrusion or fail due to filament buckling [34,35,36,37]. However, other AM technologies, such as the APF, use polymer pellets unaffected by the material’s stiffness. In this study, a specific TPO was processed through filament-based MEX and APF at different build chamber or build platform temperatures to study the influences of the print envelope temperature on the mechanical properties. The influences on the morphology of the fabricated specimen and the consequences of the infill orientation on mechanical properties in additively manufactured samples were investigated in detail. Parts printed at higher envelope temperatures were expected to have more isotropic mechanical properties. Further, more samples were printed along the Z-orientation and tested for tensile properties.

## 2. Materials and Methods

### 2.1. Material

A semi-crystalline polypropylene-ethylene copolymer with the tradename Vistamaxx^TM^ Performance Polymer 3588FL, provided by ExxonMobil Chemical Company (Houston, TX, USA), was used for this investigation. This polypropylene (PP) based on a random copolymer has a low ethylene inclusion content, resulting in a softer resin with higher impact strength as compared to the traditional homo PP. Furthermore, the material appears softer, with a Shore hardness of about 50 D. Additional material properties provided by the manufacturer are given in Table 2. The reduced crystallinity and melting temperature arising from the polymer chain arrangement generally result in better printing properties since lower processing temperatures are needed, and less shrinkage and warpage are present [29]. The material was supplied as pellets, which the freeformer can process. Filaments were produced via extrusion, as described in the following section for filament-based MEX.

### 2.2. Filament Production

Filaments were manufactured with a single screw extruder FT-20 (COLLIN Lab & Pilot Solutions GmbH, Maitenbeth, Germany) equipped with a round-shaped die. This die has an opening diameter of 1.75 mm. The extruded filament was cooled down using a water bath at room temperature. Further, the filament was pulled, measured with an optical measuring device Laser 2010 T (Sikora AG, Bremen, Germany), and finally winded onto commercially available spools on a self-developed winding unit. The machine settings for the filament extrusion are given in Table 3. The fabricated filaments were further used for the MEX 3D printing method. The rotational speed was varied from 35 to 100 min^−1^ to enhance the production speed.

### 2.3. Additive Manufacturing Process Parameters

Specimens were fabricated by APF and MEX for comparison. The chamber temperature was varied for the APF technology and the building platform temperature for the MEX method since no temperature-controlled build chamber was available. However, the printer has an enclosed build space, resulting in less air temperature fluctuations. The most important and constant specific settings are given in Table 4. These printing parameters were selected according to published recommendations. The parameter evaluation of the APF method followed a particular procedure and was performed according to Hentschel et al. [8].

For the manufacture of APF samples, an ARBURG freeformer 200-3X by ARBURG GmbH + Co. KG (Lossburg, Germany) was used. The material was deposited onto an ABS/PC compound single-use build platform held by a vacuum table. The platform generates enough adhesion to complete build jobs; however, some warping and detaching of the build platform was observed at lower temperatures. As a default, the nozzle diameter of the APF machine is 0.2 mm for depositing the droplets.

To prepare the MEX samples, a HAEG3D 140L machine provided by HAGE3D (Graz, Austria) was utilized. A unique build platform for polyolefin supplied by PPprint GmbH (Bayreuth, Germany) was used since the polypropylene adhesion to the build platform was insufficient on the standard glass platform. Mainly, at low build platform temperatures, more warping and some detachment could be observed during the process. This machine is usually fitted with a 0.4 mm nozzle, which was used for these experiments.

### 2.4. Printing Strategy

**Figure 2:**
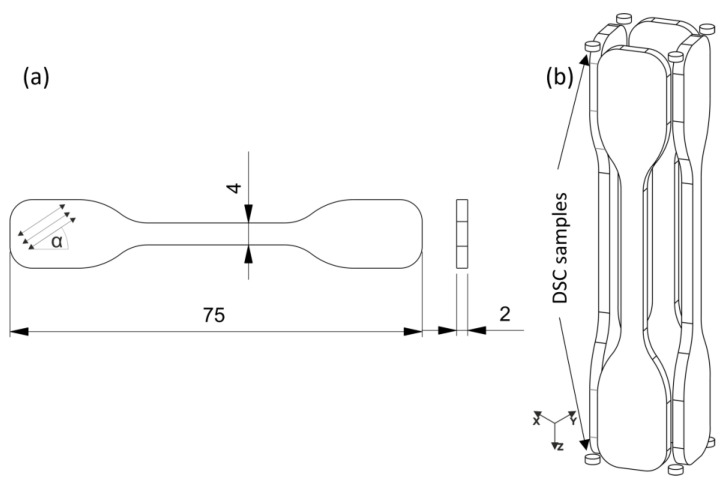
(**a**) Tensile bar according to ISO 527-5A [39] with the given dimensions in mm and the infill orientation and (**b**) configuration of tensile bars orientated in the Z-direction and DSC sample placement.

Tensile bars were printed at different processing settings to investigate the effect of the print envelope temperature on the anisotropy of 3D-printed specimens. To investigate the anisotropy, the infill angle (α) was varied from 0° to 90° and kept constant in every layer. All print envelope temperatures and the printing angles are listed in Table 5a; all setting combinations were manufactured in the first part of the study. Also, no contour line and a layer height of 0.2 mm were used for both processes when preparing the individual G-codes. The software Simplify3D v4.1 (Cincinnati, OH, USA) was used to prepare G-codes for the MEX, and the ARBURG freeformer software v2.30 (ARBURG GmbH + Co. KG) was used to define job files for the APF methods. The used geometry is shown in Figure 2a. To further study the influence of the contour lines, a combination of infill angle, contour lines, and ambient temperature at three different levels were chosen; the values are given in Table 5b.

By keeping the orientation constant, a specific anisotropic behavior is forced. Hence, the influence of print envelope temperature on anisotropy can be deduced based on these setups. Further, tensile bars were prepared in a standing manner (Z-orientation), which was only possible in the APF process due to the availability of support material. Therefore, four tensile bars were arranged in a square slightly, touching each other, as shown in Figure 2b and enclosed in solid soluble support material (ARMAT 11, ARBURG GmbH + Co. KG). Differential scanning calorimetry (DSC) samples for the thermal analysis were also printed on the building plate and the top of the construction; this is also displayed in Figure 2b. For the MEX samples, printing in Z was impossible since the construction started shaking with increasing layer numbers, resulting in a severe layer shift and failed print jobs. Using soluble support material in the MEX process was also impossible with the used equipment. The APF printed samples were then put in deionized water for around 15 h until the support material was dissolved. The samples were then dried at room temperature to prevent further post-crystallization. At least five specimens were printed and tested for each setting, except for the Z-orientation, in which only four samples were prepared.

### 2.5. Thermal Experimetal Setup 

**Figure 3:**
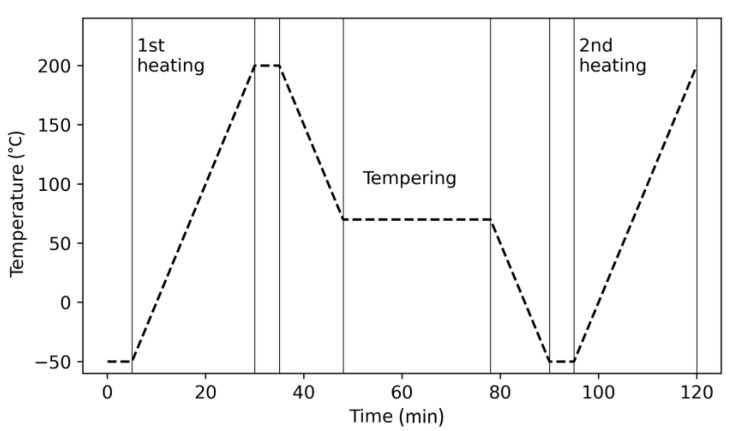
Example of temperature progress for the tempering investigations. The temperatures and times were varied for different trials.

Differential scanning calorimetry measurements were performed on a Mettler Toledo (Columbus, OH, USA) DSC1. For the standard analysis of the APF and MEX printed samples, the measurement was performed in the temperature range of −50 to 200 °C at a heating rate of 10 K/min. Further, some experiments were conducted to study the crystallization behavior of the given material. Therefore, samples cut from the virgin granules (ca. 5 mg) were tempered by holding the sample at a given temperature for a specific time using the DSC device. All analyses were performed under a nitrogen atmosphere at a flow rate of 50 mL/s to prevent any oxidation during the measurements. An example of the temperature progress over time is shown in Figure 3.

### 2.6. Microscopy

In the first step, optical microscopy (SZH; Olympus Optical Co., Tokyo, Japan) images of the central part of the specimens were taken to get an overview of the effects of the print angle and the notches developed in this way. For the investigation of the crystalline structure, cross-sectional microtome sections were cut with a Leica RM 2255 (Leica Microsystems GmbH, Wetzlar, Germany) microtome. These samples were further put onto a glass slide and wetted with a coverslip. One droplet of paraffinum liquidum with a known refractive index was placed between the samples and the glass slide. Polarized optical microscopy images were taken on the Olympus BX51 (Olympus Life Science Europe GmbH, Hamburg, Germany) at transmitted light mode. That way, influences on the morphology of the polymer at different printing parameters could be analyzed.

### 2.7. Mechanical Testing

Tensile tests were performed on a universal testing machine, Zwick Z250 (ZwickRoell GmbH + Co. KG, Ulm, Germany). Testing speed was set to 1 mm/min up to an elongation of 0.25% for the estimation of the Young’s modulus and was then increased to 50 mm/min until breakage to shorten the testing time due to the elastomeric nature of the material. The testing speeds are set according to DIN EN ISO 527-1 [40]. Mechanical clamps with riffed inserts at a defined distance of 42 mm were used to clamp the samples. Deformations were recorded at 1 FPS and evaluated by digital image correlation using a Mercury RT System (Sobriety s.r.o., Kuřim, Czech Republic). This system communicates directly with the tensile testing machine, to synchronize the deformation and load results. Therefore, a random graphite pattern was sprayed on the specimens before testing. The Young’s modulus was evaluated by the ratio of the changes in stress to the changes in strain, measured at the strain levels between 0.05 and 0.25%, respectively. For further analysis, the stresses and strains at the moment of fracture and at the yield point were evaluated. The stresses and strains shown here are engineering values only considering the initial cross section of the specimen. Correction of these values was not performed in this study.

## 3. Results and Discussion

**Figure 4:**
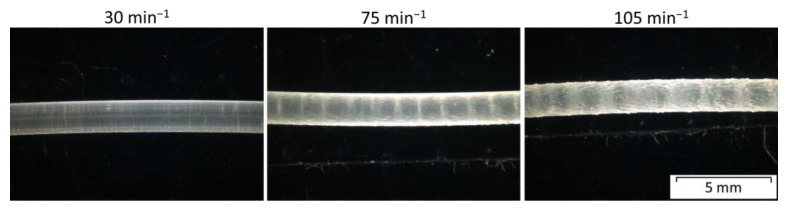
Microscopy images of the Vistamaxx^TM^ 3588FL extruded at different rotational speeds. Images were taken at a magnification of 10.

The extruded filaments, produced at a higher rotational speed, show a rougher surface, also known as the shark skin effect (Figure 4).

**Figure 5:**
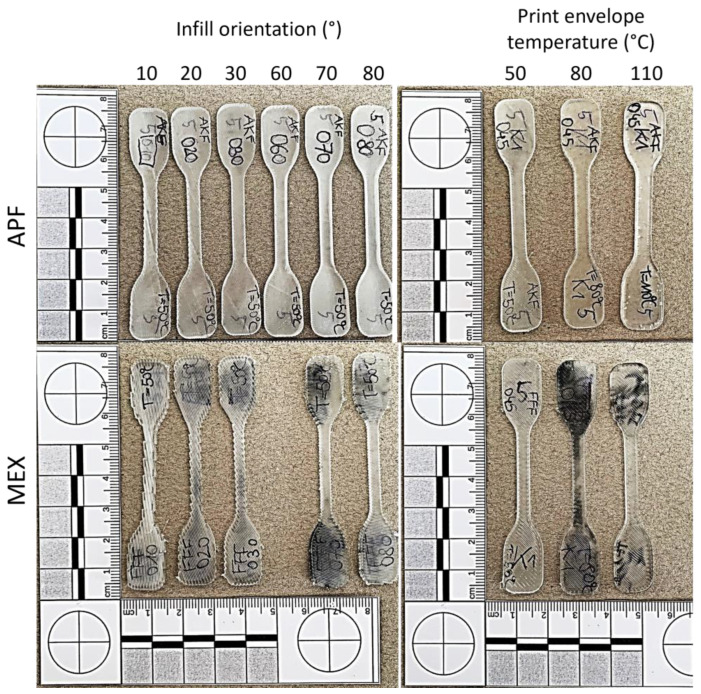
Selection of printed samples at different infill angles (**left**) and ambient temperatures (**right**) for the two additive manufacturing methods: ARBURG plastic freeforming (APF) and material extrusion (MEX).

**Figure 6:**
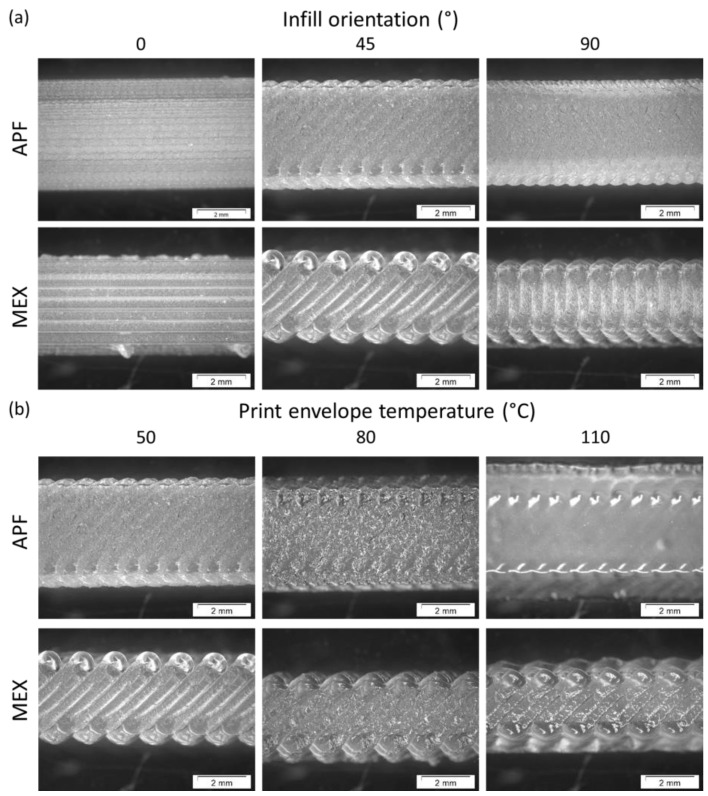
(**a**) Microscopy images of the top layers of representative samples printed at different infill angles printed at a 50 °C print envelope temperature and (**b**) samples printed with a 45° infill angle and different print envelope temperatures, using ARBURG plastic freeforming (APF) and the material extrusion (MEX) process.

The filaments produced at 30 min^−1^ show a smooth surface, as seen in the left image. For filaments made at 75 min^−1^, regular notches or weld lines appear, which indicates an unstable process resulting in a stick–slip effect or melt breaking due to the high throughput and wall slip. After increasing the rotational speed further, a clear example of the shark skin effect can be seen. Based on these results, only filaments produced at the lowest rotational speed were used for further experiments. Since the shark skin effect occurred during the filament extrusion process at a higher extrusion speed, it could also happen during MEX processing at higher speeds [10]. Therefore, the processing speed was limited, and no shark skin was visible on the printed parts (Figure 5 and Figure 6).

All parts were printed without any special visible errors on either machine. Only the samples printed at low print envelope temperatures show low bed adhesion and thus some warping. An overview of selected printed parts is displayed in Figure 5, to give an example.

As one can see, samples printed at different ambient temperatures show different appearances. The specimens, printed on the freeformer at a 110 °C chamber temperature, are more transparent but show some voids inside the specimens (see Figure 6b: APF). The black-colored parts on the samples (particularly for MEX) are residuals of the building plate, which were not removeable from the samples. However, such residuals are primarily present on the surface of the clamping areas of the specimens, and hence, do not influence the measurements. Furthermore, the edges of the samples are not smooth for some of the samples since no contour line was used. Especially, the MEX printed samples show predominant notches (Figure 6a: MEX 45° and 90°). For the APF printed samples, notches are neglectable, and even the individual strand lines tend to vanish at elevated ambient temperatures (Figure 6b: APF) and with increasing infill angles (Figure 6a: APF 45° and 90°).

Comparing the two manufacturing methods, the strands of the APF samples are narrower than for MEX. This is due to the smaller nozzle size used as a standard for APF. The notches in the MEX samples may further lead to a weaker performance of the specimens.

### 3.1. Thermal Analysis

**Figure 7:**
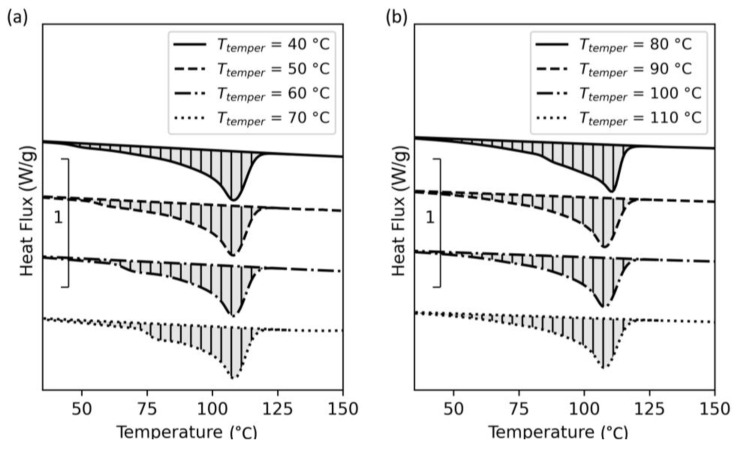
DSC results of tempering trials performed within the DSC device for specimens printed via ARBURG plastic freeforming (APF) at different tempering temperatures: (**a**) 40 to 70 °C and (**b**) 80 to 110 °C.

To understand the influences of an isothermal holding temperature and time during cooling of the polymer, DSC experiments as described in Section 2.5 were performed. The results of these tempering experiments are shown in Figure 7.

**Figure 8:**
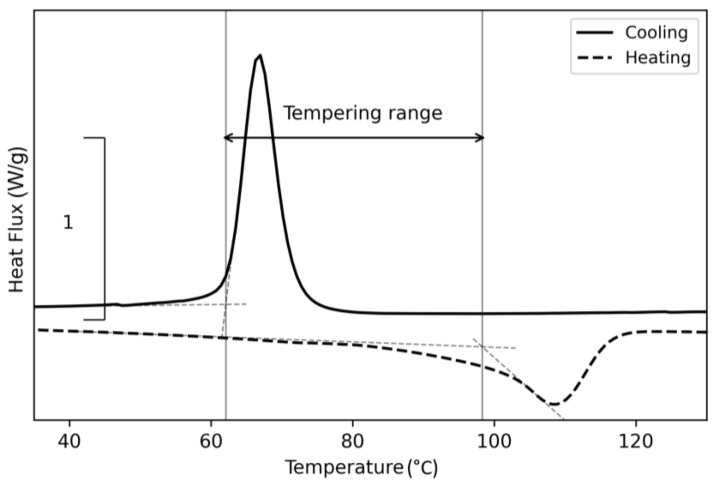
Discovered effective tempering range of the Vistamaxx^TM^ 3588FL, based on the results from the tempering trials from 40 to 110 °C.

Comparing the individual curves, differences in the heating curves start to occur at a 60 °C holding temperature and vanish at holding temperatures over 90 °C, which correlates with the onset of the cold crystallization function and the onset of the melting peak for this copolymer. These secondary peaks might indicate the existence of a different crystalline structure due to longer crystallization time. For demonstration, the effective range between the measured onsets at 62 and 97 °C, respectively, is displayed in Figure 8.

The peak temperatures of the results shown in Figure 7 are similarly to virgin polypropylene (Figure 8), and the selected temperature range shows similarities to the crystalline mobility temperature range mentioned by Fiebig et al. [41]. The recommended annealing temperature for PP is 85 °C [42], which can also be applied for this copolymer due to its low ethylene content. A further increase in ethylene content leads to a further reduction in melting temperature [43]. Hence, the print envelope temperatures are selected based on these results to be below, in between, and above this range.

**Figure 9:**
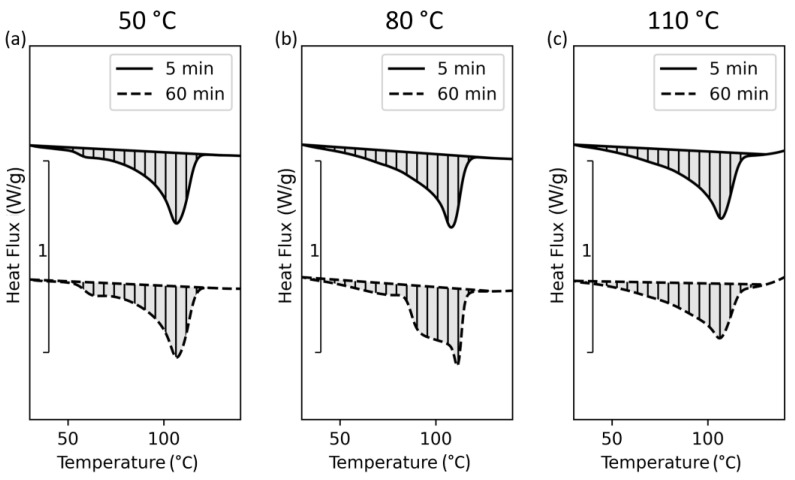
DSC results of tempering trials performed within the DSC device for the material in pellets formed at different times and tempering temperatures: (**a**) 50 °C, (**b**) 80 °C, and (**c**) 110 °C.

Further DSC experiments were conducted to study the influence of the tempering time on crystallinity. Unlike the previous experiments, this study was performed on pellets at different isothermal holding times and at only three different temperature levels. These results are presented in Figure 9.

The resulting curves show a significant difference in the melting functions of the samples holding at 80 °C at different times. This indicates the time dependency of the crystallization kinetics and refers to a secondary (β) crystalline structure which can be triggered at a defined undercooling and isothermal holding [44]. Because of the time dependence and the quite slow processing speed in AM, different crystalline structures can occur along the Z-direction of a 3D-printed part.

**Figure 10:**
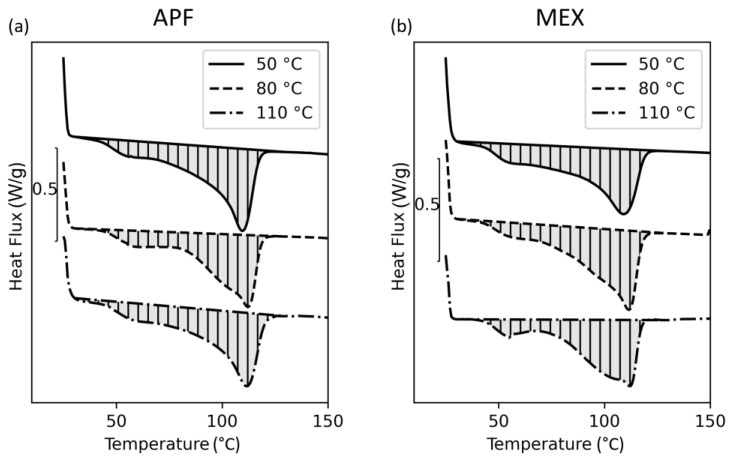
DSC results of specimens printed via (**a**) ARBURG plastic freeforming (APF) and (**b**) material extrusion (MEX) at different envelope temperatures according to the standard measuring procedure. The melting areas for the calculation of the melting enthalpy are displayed in grey.

DSC analyses were also performed on printed parts. The resulting curves of the first heating cycle measured on samples taken from samples printed via APF and MEX are presented in Figure 10.

Only the first heating curve of samples processed at 80 °C ambient temperature in APF and at 110 °C in MEX show a slightly different behavior at similar settings for the print envelope temperatures. These results also lead to the assumption of a different crystalline structure caused by elevated print envelope temperatures. However, this effect is diminished by even higher temperatures for APF. The secondary melting peak at around 60 °C may be caused by fast cooling during the processes. A similar melting peak occurs in the sample tempered at 50 °C, which may also indicate insufficient cooling due to a low heat conductivity of the polymer, and hence, residual heat within the printed part.

**Figure 11:**
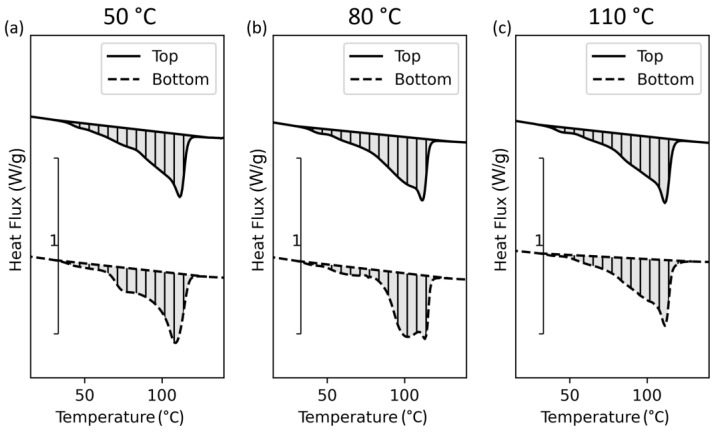
DSC results of tempering trials performed within the DSC device for specimens printed via ARBURG plastic freeforming (APF) at different print heights/times and tempering temperatures: (**a**) 50 °C, (**b**) 80 °C, and (**c**) 110 °C.

Tensile bars were printed standing in the Z-orientation, with DSC samples placed at the bottom and on the top (Figure 2b). The results of the DSC analysis are given in Figure 11.

**Figure 12:**
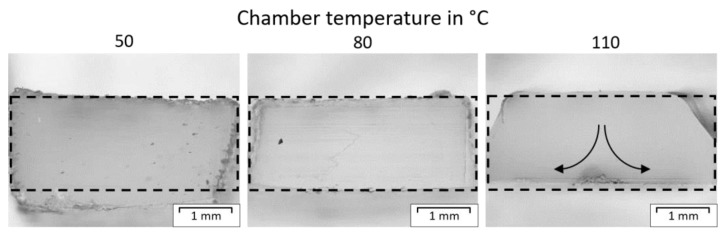
Cross section of tensile bars printed at different chamber temperatures with the APF and indicated flow direction on the right figure.

One can spot similar differences as observed within the printed samples. Hence, the tempering time significantly influences the crystalline structure, resulting in anisotropy. Thus, if parts with a high expansion in the Z-direction are printed at evaluated temperatures, the time the polymer is exposed to this environment changes from top to bottom. Nevertheless, a more homogeneous crystallinity can be observed if printed at a higher ambient temperature, but the material is still molten. Therefore, the form stability is low and gets lower at rising temperatures due to decreased zero viscosity and melt strength. Further, the polymer starts to flow due to gravity. This flow results in high differences in shape and low accuracy, as seen in Figure 12. Therefore, a compromise between geometrical accuracy and mechanical strength must be found.

However, even if the appearance of the cross sections did change, the overall mass of the samples, and hence, size did not change at different temperatures. Also, the trace width did not change with temperature, but as mentioned above, material flow can be present at higher temperatures and lead to deviations in the cross sections.

### 3.2. Polarization Microscopy

**Figure 13:**
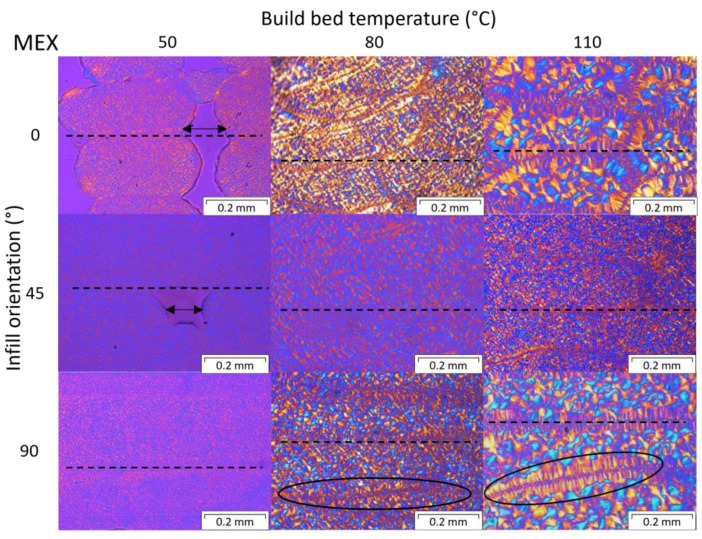
Polarized optical microscopy of samples printed via material extrusion (MEX) at 0°, 45°, and 90° infill angles and 50, 80, and 110 °C bed temperatures. Visible layer lines are marked by dashed lines, shish kebab structures are circled in, and voids are shown by the arrows.

**Figure 14:**
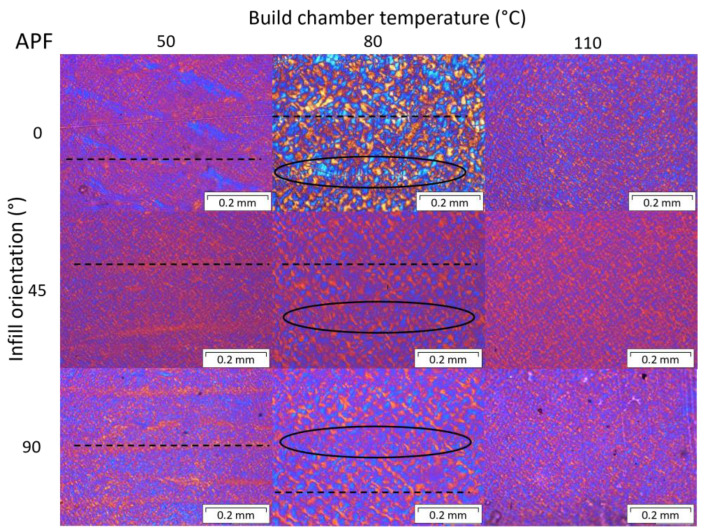
Polarized optical microscopy of specimens prepared by ARBURG plastic freeforming (APF) at different print orientations and bed temperatures. Visible layer lines are marked by dashed lines and shish kebab structures are circled in.

Polarized optical microscopy was performed at the cross sections of the 0°, 45°, and 90° samples, fabricated at different print envelope temperatures. Figure 13 shows the crystalline structures of the MEX printed samples and Figure 14 for APF specimens.

One can see the differences in the crystalline structures for different build bed temperatures and print orientations. At higher temperatures, larger spherulites are formed, and so-called shish kebab structures (black ovals) get more and more present around the connection areas of the individual strands, as similarly observed by Petersmann et al. [22]. Fischer et al. [45] also show increased spherulite size at higher isothermal holding temperatures and longer holding times. This larger size of the crystalline structures is probably due to the higher temperature and prolonged crystallization period (i.e., more time is available to grow before solidification). However, the individual layers and strands are still visible in all samples (dashed lines), even more at higher temperatures due to the formed shish kebab structures. Further, some voids are present in samples printed at low bed temperatures. These voids might be due to under-extrusion during the build process or fast cooling and shrinking, hindering the fusion of the individual strands. Voids can lead to a loss in strength and high scattering in the mechanical testing results.

For the APF printed samples, layer lines (dashed lines) can be observed at a chamber temperature of 50 °C and in the 80 °C samples, even though they are not as visible as in the MEX samples. Here as well, shish kebab crystalline structures (black ovals) can be detected in samples printed with an 80 °C build chamber temperature. However, the polarized optical microscopy images of samples printed at 110 °C show a homogenous crystallinity, without distinguishable weld lines. This is an indication of a homogeneous material behavior, when printed at higher envelope temperatures. By looking at the figure of the sample printed at 80 °C and a 0° infill orientation, a similar crystalline structure on the bonding area can be observed as in the MEX samples printed at 110 °C only at smaller sizes. The smaller size might be due to the thinner strands or droplets deposited with the APF compared to the MEX technology. The internal temperatures of the MEX printed samples may be a little lower, compared to the APF printed parts, since only the platform was heated in MEX. This can also be observed from the DSC results (Figure 10), where the heating curves of samples printed at 80 °C with APF are comparable with those of samples printed at 110 °C with MEX.

### 3.3. Tensile Properties

**Figure 15:**
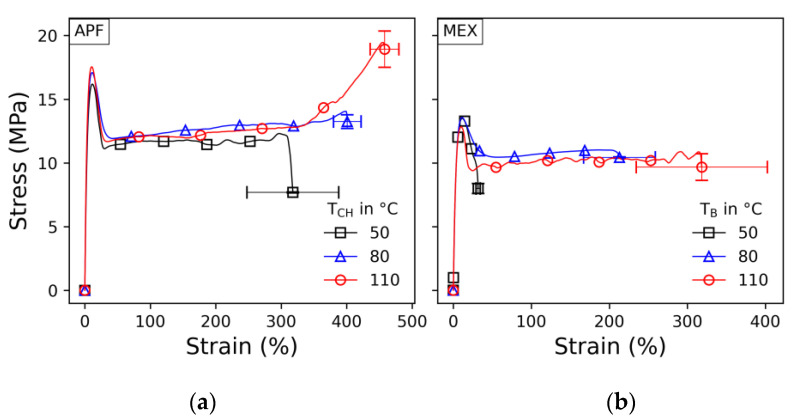
Stress–strain curves of samples printed with no contour lines and a 45° infill angle at different print envelope temperatures for (**a**) APF and (**b**) MEX.

Selected engineering stress–strain curves are shown in Figure 15 with their standard error of the break values. Note that these results were evaluated based on the initial cross section. Only the APF samples printed at a 110 °C chamber temperature show a hyperplastic increase at high elongation; all other presented samples break in the plateau. Hence, better bonding was achieved in these settings. The yield strength and evaluation of the stress–strain curves are similar for all other parameters.

In comparison, samples printed with the APF show a higher stress level than those printed with MEX. A higher necking behavior can also be observed, but the plateaus show almost the same stress levels. As mentioned, voids may be present more in the MEX samples than in the APF samples, especially at lower print envelope temperatures, which might explain the lower stress and strain values.

**Figure 16:**
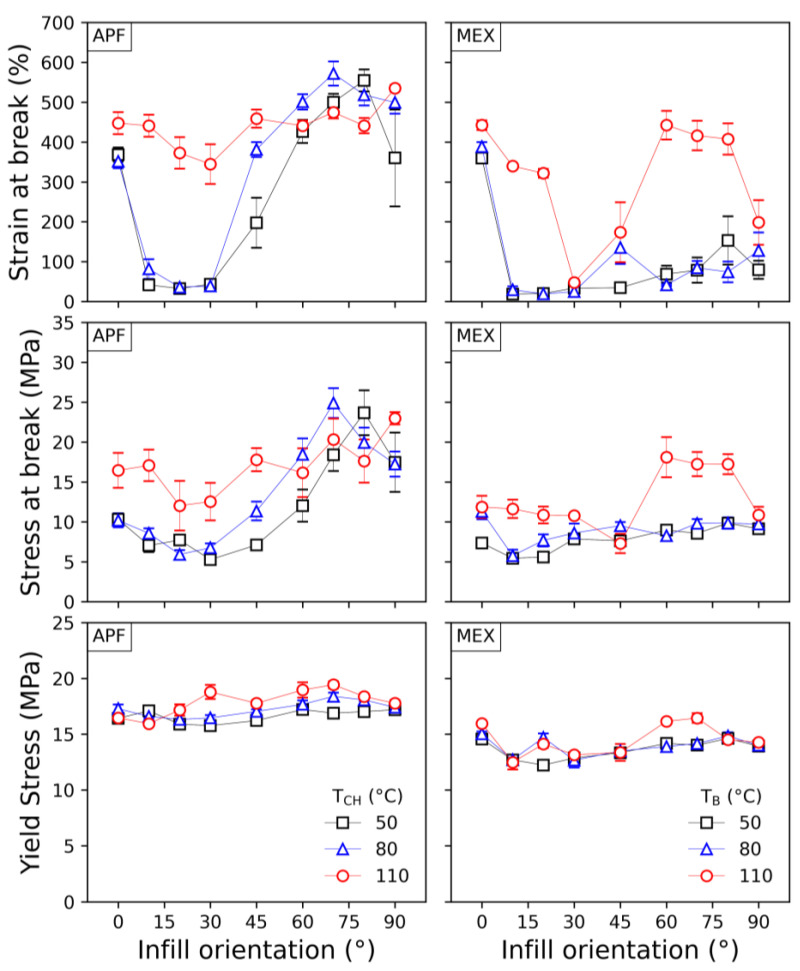
Tensile properties of samples printed via ARBURG plastic freeforming (APF) at different chamber temperatures (T_CH_) and material extrusion (MEX) at different print bed temperatures (T_B_) and with infill angles.

The results of the tensile tests from samples printed at different infill angles are displayed in Figure 16. The resulting yield stresses and strains at break values are plotted over the infill angles set for the specimens (for more information, the results are listed in Table S1).

The yield strengths of the samples are constant within a specific range, while the strain at break values alternate along the different orientations, particularly at lower print envelope temperatures. The yield strength only shows minor variances, which may be due to the ductile nature of the material. A drop in tensile strength over the infill angle was expected, similarly to the results of Dudescu et al. [46]. However, by comparing the different print envelope temperatures, one can see the effect of the infill orientation lowering at 110 °C, making the parts more isotropic in such a manner. Also, no significant changes in strain at break were shown in the mentioned investigation [46] in contrast to the results obtained in this study. The increasing strain at break values for MEX samples printed at a 110 °C bed temperature and infill angles of 70°, 80°, and 90° compared to samples printed at lower infill angles may be due to a better intralayer bonding of the individual strands. At higher infill angles, the time between the next strand laid to the previous shortens significantly, resulting in better welding [22,24,26,47]. Similar behavior can be seen for the APF printed parts but at all temperature levels. In contrast to the literature, no significant better mechanical performance was detected [48,49].

Furthermore, the APF samples show higher values at a 90° infill angle than at 0°, which might have been the case due to better bonding between adjacent strands caused by the shorter travel distance, hence higher residual temperatures due to less time for cooling. A similar result was observed by Charlon et al. [12] and Ramezani Dana et al. [50] for ABS. The highest impact of the print envelope temperature was observed at the strain at break values. At this stage, the specimen is highly elongated; hence, minor imperfections can lead to ultimate failure. Thus, a higher scattering can be seen in the results from the strain and stress at break values. It can also indicate the best bonding for the low infill angle specimens for the APF method and samples printed at a 110 °C bed temperature with the MEX method.

**Figure 17:**
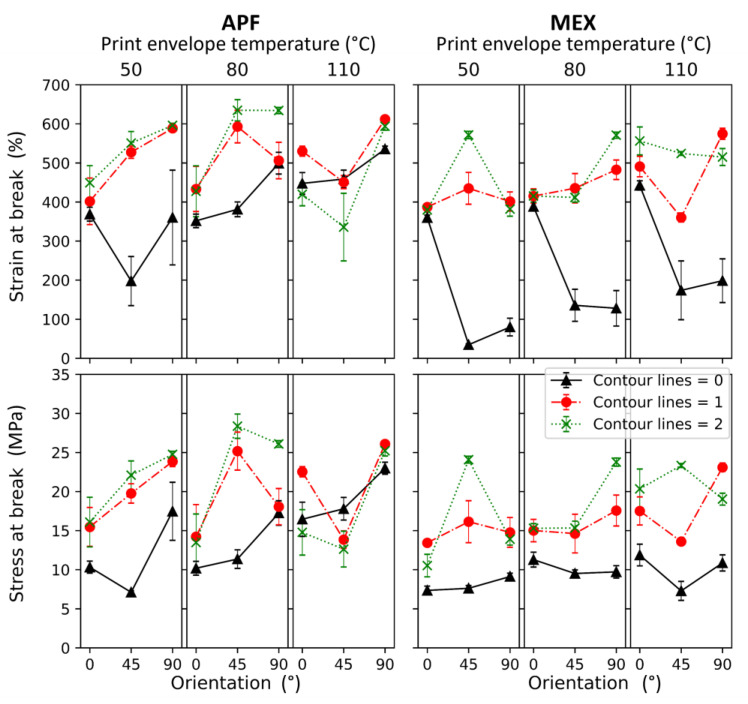
Tensile properties of samples printed via ARBURG plastic freeforming (APF) and material extrusion (MEX) at different envelope temperatures, infill angles, and contour lines.

Nevertheless, additively manufactured samples are mostly printed with a specific number of contour lines since it helps to achieve a better visual appearance (i.e., no jagged edges). According to Table 5b, more samples were prepared and tested to determine the influence of this feature on mechanical properties. The results are shown in Figure 17 and are further listed in Tables S2 and S3 for more details.

Here again, APF samples printed at a 90° infill angle show higher stress and strain values than APF samples printed at lower angles overall. The samples printed with zero contour lines show the lowest values for both methods. In the case of the APF process, this was also observed by Charlon et al. [12]. Further, the effect of contour lines vanishes for the APF samples printed at a 110 °C chamber temperature, and a drop for the samples printed with a 45° infill angle appears. This result might arise from a more considerable void concentration at the border between the infill and contour, resulting from the infill path orientation. These voids can more easily develop at a higher chamber temperature, leading to lower stress and strain results at break. The formation of such voids or micro voids was also discussed by Hentschel et al. [51]. Another explanation for the slight increase in stress at break might be reduced internal stress. Samy et al. [27] found a reduction of up to 2 MPa in internal stress when PP was printed at the evaluated ambient temperatures. Besides those effects, no significant patterns could be observed. However, it must be highlighted that the highest mechanical performance for both methods was reached at an infill angle of 45° and with two contour lines, which was also concluded by Hirsch et al. [14]. These fabrication strategies correspond with the recommended setting for 3D printing, at least for MEX methods. Furthermore, the differences in the results between non- and single-contour line samples led to the assumption that notches cause a significant drop in performance in angled samples. Due to the ductile nature of the polymer, this has a higher influence on stress and strain at break than on yield strength.

**Figure 18:**
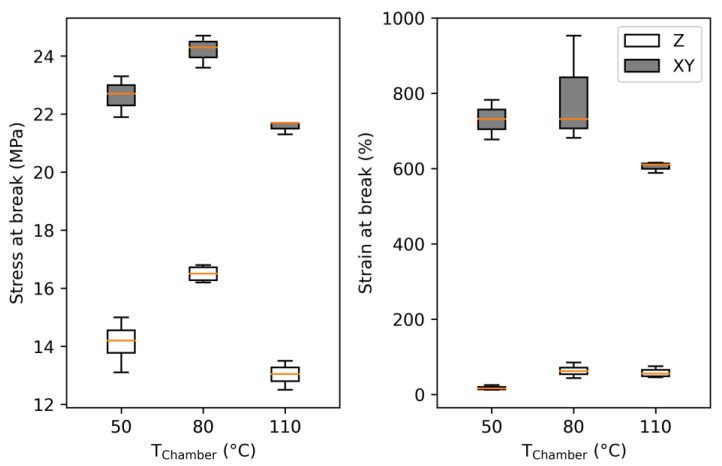
Tensile results of ARBURG plastic freeforming (APF) samples printed in the Z-orientation at different chamber temperatures and compared to XY-orientated samples.

To further prove decreased anisotropy and higher interlayer adhesion, tensile samples were printed standing in the Z-orientation at different chamber temperatures. As mentioned, it was not possible to print them with the MEX technology since the material is too soft and started to wobble with progressing height. Further, it was not possible to use support material due to hardware limitations. The results of these tensile tests are displayed in box plots in Figure 18.

Regardless of the chamber temperature, the stress and strain at break for the specimens printed vertically (Z-orientation) were significantly lower than those printed horizontally (XY-orientation), as other researchers observed [14,52,53].

The results of samples printed at a 80 °C chamber temperature are way below the stresses and strains observed in the previous samples. Most significantly, the strain values dropped dramatically, resulting in a less ductile behavior. However, the values for stress at break measured for samples printed at 50 °C show very high scattering. Samples printed at the highest chamber temperatures performed the worst, which may have been the result of poor printing accuracy due to high material flow. These samples also had a rough surface, which could have acted as notches and drastically reduced the stress and strain at break values. Hence, thin samples or features printed vertically with this polymer should be printed at room or even lower chamber temperatures for better layer stacking and, thus, better layer adhesion. 

Further optimization can be performed by optimizing the printing speed of the APF method, increasing the heating time, and reducing the acceleration forces acting on the printed samples. Interlayer diffusion can be enhanced at longer diffusion times or crystallization times. More extended diffusion can be realized at higher chamber temperatures or slower processing speeds. The results show that higher chamber temperatures lead to weaker samples; thus, slower processing might produce better results. However, in-depth optimization of the diffusion, coalescence, and crystallization may further improve isotropic material behavior.

## 4. Conclusions

In a nutshell, additively manufactured parts with the use of polyolefin show anisotropy in mechanical behavior and morphology, among other properties, due to the generative addition of material and thermal history. Among other machines and process parameters, the environment of printing significantly matters. Especially for semi-crystalline materials, the print envelope temperature can strongly influence the morphology and resulting material properties. The idea behind this study was to generate an isotropic additively manufactured part by evaluating print envelope temperatures in two corresponding processes, namely material extrusion (MEX) and ARBURG plastic freeforming (APF). The main conclusions of this study can be summarized as follows:

- A different crystalline structure in samples printed at 80 °C by APF and 110 °C by MEX was observed, and a time dependency was shown for the formation of a secondary crystalline structure.
- Weld lines between individual layers or even strands or droplets were no longer present, resulting in a homogeneous crystallinity over the cross –section, when a chamber temperature of 110 °C was used in the APF process. This observation leads to the assumption that a more isotropic behavior can be expected at this chamber temperature.
- The yield strength of samples printed with different infill angles and at different envelope temperatures shows no significant difference. However, stress and strain values at break show an influence of the infill angles, which diminishes at higher print envelope temperatures. Hence, intralayer bonding can be improved by increasing the print envelope temperatures.
- At higher envelope temperatures, crystallization times are prolonged, leading to better welding, resulting in a higher isotropic mechanical behavior for samples printed at higher print envelope temperatures. However, isotropy was not observed in samples printed along the Z-direction. The samples printed at an 80 °C chamber temperature showed the best performance but were still significantly lower than samples prepared in the XY-direction.
- APF specimens showed better overall mechanical performance than MEX samples, most likely due to the lower porosity obtained by the APF method [54]. Since the chamber of the MEX machine was not actively heated, a similar morphology could be observed but at higher print build platform temperatures. The influence of the infill orientation remains similar for APF and MEX.
- The effect of contour lines on mechanical properties was similar for MEX and APF processes. It was found that tensile properties peaked at a 45° infill with two contour lines but at different ambient temperatures for the two evaluated processes.
- At higher envelope temperatures, the TPO material was partially molten during printing, and hence, the material tended to flow, resulting in low geometrical stability. Therefore, the envelope temperature must be reduced if the printing time is extended, or high geometrical accuracy is needed. As a recommendation, the print envelope temperature should be kept in the range of the cold crystallization and the melting onset (i.e., 62 and 97 °C).

## 5. Future Work

DMA analysis can be performed to better understand crystallinity, phase formations, and influences on segmentation behavior [55]. This characterization might also be interesting when applying similar approaches to materials with higher ethylene content, which are commercially available. However, regarding 3D printing, the selected TPO might already be at the cutting edge of reliable printability, but others might be interesting for research because their melting temperatures and crystallinity decrease with increased ethylene content [32]. The printability of these copolymers is planned in future works. Also, a study on the diffusion and coalescence properties could further improve mechanical properties, especially in the Z-orientation, as proposed by Beniè et al. [24]. 

## Figures and Tables

**Table 1:** List of key parameters for the APF [12] and MEX [9] processes.

APF	MEX
Nozzle temperature	Nozzle temperature
Chamber temperature	Bed temperature
Discharge parameter	Nozzle diameter
Drop aspect ratio	Layer height
Layer height	Tarce width
Infill density	Infill density
Infill orientation	Infill overlap
Part orientation	Infill orientation
Contour overlap	Part orientation
Printing speed	Printing speed

**Table 2:** Material properties of Vistamaxx^TM^ 3588FL provided by the supplier [38].

Property	Value	Unit
Density	0.889	g/cm^3^
Melt Mass Flow Rate (230 °C/2.16 kg)	8	g/10 min
Hardness (Shore)	50	D
Tensile Yield Strength	16	MPa
Tensile Stress at Break	26	MPa
Tensile Strain at Break	637	%

**Table 3:** Extrusion processing conditions for the filament production of Vistamaxx^TM^ 3588FL.

Parameter	Value	Unit
Temperature Zone 1	190	°C
Temperature Zone 2	195	°C
Temperature Zone 3	200	°C
Screw Speed	35–105	min^−1^

**Table 4:** Processing parameters for the APF and MEX processes.

APF			MEX		
Parameter	Value	Unit	Parameter	Value	Unit
Nozzle Temperature	220	°C	Nozzle Temperature	220	°C
Screw Speed	5	min^−1^	Printing Speed	20	mm/min
Drop Aspect Ratio	1.50	–	Filament Diameter	1.75	mm
Chamber Temperature	50, 80, 110	°C	Bed Temperature	50, 80, 110	°C

**Table 5:** (**a**) Print strategies for the APF and MEX samples to study the influence of infill orientation and print envelope temperature without contour line and (**b**) the second setup to include a specified number of contour lines but only three angles to reduce the number of specimens.

(**a**)									
**Print Envelope Temperature in °C**	50				80		110		
**Orientation in °**	0	10	20	30	45	60	70	80	90
(**b**)									
	**Print Envelope Temperature in °C**				**Orientation in °**		**Number of Contour Lines**		
Low	50				0		0		
Middle	80				45		1		
High	110				90		2		

## Data Availability

Data is contained within the article or Supplementary Materials.

## Supplementary Materials

The following supporting information can be downloaded at: https://www.mdpi.com/article/10.3390/polym15183785/s1, Table S1: Tensile test results from the samples printed at different infill orientations and print envelope temperatures. For a comparison stresses at break (a), strain at break (b) and yield stresses (c) are listed here; Table S2: Tensile results for the specimen printed with the APF method at different infill orientations, chamber temperatures and number of contour lines. Values for stresses at break (a) and Strain at break (b) are listed; Table S3: Tensile results for the specimen printed with the MEX method at different infill orientations, chamber temperatures and number of contour lines. Values for stresses at break (a) and Strain at break (b) are listed.
