# Supplementary material for: Influence of the Print Envelope Temperature on the Morphology and Tensile Properties of Thermoplastic Polyolefins Fabricated by Material Extrusion and Material Jetting Additive Manufacturing
Source: Polymers (Basel). 2023 Sep 16;15(18):3785. doi: 10.3390/polym15183785 (PMC10534743; doi:10.3390/polym15183785)
Supplement: Supplementary file 1 [file polymers-15-03785-s001.zip › polymers-2579186-supplementary.pdf]

polymers

MDPI

# Supplementary Information

# Influence of the Print Envelope Temperature on the Morphology and Tensile Properties of Thermoplastic Polyolefins Fabricated by Material Extrusion and Material Jetting Additive Manufacturing

**Lukas Hentschel [1], Sandra Petersmann [2,†], Frank Kynast [3], Ute Schäfer [4,5], Clemens Holzer [1] and Joamin Gonzalez-Gutierrez [1,*,‡]**

**Table S1.** Tensile test results from the samples printed at different infill orientations and print envelope temperatures. For a comparison stresses at break (a), strain at break (b) and yield stresses (c) are listed here.

| a) | Stress at break (MPa) | | | | | |
|---|---|---|---|---|---|---|
| | | APF | | | MEX | |
| | 50 °C | 80 °C | 110 °C | 50 °C | 80 °C | 110 °C |
| 0° | 10.3 ± 0.8 | 10.2 ± 0.9 | 16.5 ± 2.2 | 7.3 ± 0.5 | 11.3 ± 1 | 11.9 ± 1.4 |
| 10° | 7 ± 0.8 | 8.6 ± 0.6 | 17.1 ± 2 | 5.4 ± 0.2 | 5.8 ± 0.8 | 11.6 ± 1.1 |
| 20° | 7.7 ± 0.4 | 5.9 ± 0.5 | 12 ± 3.1 | 5.6 ± 0.1 | 7.7 ± 0.7 | 10.9 ± 1.1 |
| 30° | 5.3 ± 0.5 | 6.7 ± 0.5 | 12.5 ± 2.3 | 7.9 ± 0.5 | 8.6 ± 1.2 | 10.8 ± 0.3 |
| 45° | 7.1 ± 0.2 | 11.4 ± 1.2 | 17.8 ± 1.4 | 7.6 ± 0.4 | 9.5 ± 0.5 | 7.3 ± 1.2 |
| 60° | 12 ± 2 | 18.5 ± 2 | 16.2 ± 3.1 | 9 ± 0.4 | 8.3 ± 0.5 | 18.1 ± 2.5 |
| 70° | 18.4 ± 2 | 24.9 ± 1.9 | 20.3 ± 2.6 | 8.6 ± 0.5 | 9.8 ± 0.5 | 17.2 ± 1.5 |
| 80° | 23.7 ± 2.8 | 20 ± 1.9 | 17.6 ± 2.7 | 9.8 ± 0.5 | 9.8 ± 0.7 | 17.2 ± 1.3 |
| 90° | 17.5 ± 3.7 | 17.2 ± 1.6 | 23 ± 0.8 | 9.1 ± 0.4 | 9.7 ± 0.8 | 10.9 ± 1 |
| b) | Stain at break (%) | | | | | |
| | | APF | | | MEX | |
| | 50 °C | 80 °C | 110 °C | 50 °C | 80 °C | 110 °C |
| 0° | 369 ± 18 | 352 ± 17 | 447 ± 28 | 360 ± 6 | 388 ± 12 | 442 ± 12 |
| 10° | 42 ± 5 | 82 ± 24 | 441 ± 28 | 19 ± 0.5 | 30 ± 8 | 340 ± 4 |
| 20° | 33 ± 5 | 36 ± 2 | 373 ± 40 | 20 ± 2 | 19 ± 2 | 322 ± 11 |
| 30° | 43 ± 11 | 39 ± 2 | 345 ± 50 | 34 ± 8 | 25 ± 11 | 48 ± 4 |
| 45° | 198 ± 63 | 381 ± 19 | 459 ± 23 | 35 ± 2 | 135 ± 41 | 174 ± 75 |
| 60° | 427 ± 29 | 501 ± 19 | 441 ± 13 | 69 ± 21 | 42 ± 2 | 442 ± 36 |
| 70° | 500 ± 21 | 572 ± 30 | 474 ± 15 | 79 ± 31 | 84 ± 17 | 416 ± 37 |
| 80° | 555 ± 28 | 518 ± 27 | 441 ± 19 | 153 ± 61 | 74 ± 26 | 408 ± 39 |
| 90° | 360 ± 121 | 499 ± 28 | 535 ± 8 | 80 ± 23 | 128 ± 45 | 198 ± 56 |
| c) | Yield stress (MPa) | | | | | |
| | | APF | | | MEX | |
| | 50 °C | 80 °C | 110 °C | 50 °C | 80 °C | 110 °C |
| 0° | 16.4 ± 0.3 | 17.3 ± 0.4 | 16.5 ± 0.2 | 14.6 ± 0.2 | 15 ± 0.2 | 16 ± 0.2 |
| 10° | 17.1 ± 0.2 | 16.6 ± 0.1 | 15.9 ± 0.2 | 12.7 ± 0.1 | 12.7 ± 0.2 | 12.5 ± 0.6 |
| 20° | 15.9 ± 0.4 | 16.3 ± 0.1 | 17.2 ± 0.5 | 12.2 ± 0.2 | 14.7 ± 0.4 | 14.1 ± 0.3 |
| 30° | 15.8 ± 0.5 | 16.5 ± 0.3 | 18.8 ± 0.6 | 12.9 ± 0.1 | 12.7 ± 0.7 | 13.2 ± 0.2 |

| | | | | | | |
|---|---|---|---|---|---|---|
| 45° | 16.2 ± 0 | 17 ± 0.2 | 17.8 ± 0.3 | 13.3 ± 0.2 | 13.5 ± 0.1 | 13.4 ± 0.8 |
| 60° | 17.2 ± 0.4 | 17.7 ± 0.4 | 19 ± 0.7 | 14.2 ± 0.1 | 13.9 ± 0.2 | 16.1 ± 0.2 |
| 70° | 16.9 ± 0.4 | 18.4 ± 0.3 | 19.4 ± 0.3 | 14 ± 0.1 | 14.2 ± 0.3 | 16.4 ± 0.4 |
| 80° | 17 ± 0.2 | 18.1 ± 0.1 | 18.4 ± 0.2 | 14.6 ± 0.1 | 14.8 ± 0.3 | 14.5 ± 0.2 |
| 90° | 17.2 ± 0.3 | 17.4 ± 0.1 | 17.8 ± 0.3 | 13.9 ± 0.2 | 14 ± 0.1 | 14.3 ± 0.2 |

**Table S2.** Tensile results for the specimen printed with the APF method at different infill orientations, chamber temperatures and number of contour lines. Values for stresses at break (a) and Strain at break (b) are listed.

| **a)** | | **Stress at break (MPa)** | | |
|---|---|---|---|---|
| | Chamber temperature → | 50 °C | 80 °C | 110 °C |
| Orientation ↓ | Number of contour lines ↓ | | | |
| 0° | 0 | 10.3 ± 1.7 | 10.2 ± 2 | 16.5 ± 4.9 |
| | 1 | 15.5 ± 5.6 | 14.2 ± 9.2 | 22.5 ± 1.5 |
| | 2 | 16.1 ± 7.1 | 13.5 ± 8.2 | 14.8 ± 6.5 |
| 45° | 0 | 7.1 ± 0.5 | 11.4 ± 2.6 | 17.8 ± 3.2 |
| | 1 | 19.8 ± 2.8 | 25.2 ± 5.4 | 13.9 ± 2.5 |
| | 2 | 22.1 ± 4.1 | 28.4 ± 3.5 | 12.6 ± 5.1 |
| 90° | 0 | 17.5 ± 8.3 | 17.2 ± 3.5 | 23 ± 1.7 |
| | 1 | 23.8 ± 1.5 | 18.1 ± 5.2 | 26.1 ± 0.9 |
| | 2 | 24.8 ± 0.9 | 26.1 ± 1 | 25.2 ± 1.5 |
| **b)** | | **Strain at break (MPa)** | | |
| | Chamber temperature → | 50 °C | 80 °C | 110 °C |
| Orientation ↓ | Number of contour lines ↓ | | | |
| 0° | 0 | 369 ± 40 | 352 ± 39 | 447 ± 62 |
| | 1 | 402 ± 133 | 433 ± 129 | 530 ± 28 |
| | 2 | 449 ± 98 | 427 ± 145 | 420 ± 67 |
| 45° | 0 | 198 ± 140 | 381 ± 42 | 459 ± 51 |
| | 1 | 527 ± 35 | 593 ± 93 | 451 ± 38 |
| | 2 | 551 ± 66 | 635 ± 61 | 336 ± 193 |
| 90° | 0 | 360 ± 271 | 499 ± 62 | 535 ± 17 |
| | 1 | 589 ± 8 | 506 ± 104 | 612 ± 12 |
| | 2 | 596 ± 12 | 634 ± 18 | 594 ± 23 |

**Table S3.** Tensile results for the specimen printed with the MEX method at different infill orientations, chamber temperatures and number of contour lines. Values for stresses at break (a) and Strain at break (b) are listed.

| **a)** | | **Stress at break (MPa)** | | |
|---|---|---|---|---|
| | Chamber temperature → | 50 °C | 80 °C | 110 °C |
| Orientation ↓ | Number of contour lines ↓ | | | |
| 0° | 0 | 7.3 ± 1.2 | 11.3 ± 2.1 | 11.9 ± 3.1 |
| | 1 | 13.4 ± 1 | 15 ± 3.2 | 17.5 ± 4 |
| | 2 | 10.5 ± 3.2 | 15.3 ± 1.3 | 20.3 ± 5.7 |
| 45° | 0 | 7.6 ± 0.8 | 9.5 ± 1 | 7.3 ± 2.7 |
| | 1 | 16.1 ± 6 | 14.6 ± 5.5 | 13.6 ± 0.5 |

| | | | | |
|---|---|---|---|---|
| | 2 | 24.1 ± 1 | 15.3 ± 2 | 23.3 ± 0.6 |
| | 0 | 9.1 ± 1 | 9.7 ± 1.8 | 10.9 ± 2.3 |
| 90° | 1 | 14.8 ± 4.3 | 17.6 ± 4.4 | 23.1 ± 1.2 |
| | 2 | 13.9 ± 1.7 | 23.8 ± 1.2 | 19 ± 1.7 |

| **b)** | | **Strain at break (MPa)** | | |
|---|---|---|---|---|
| Chamber temperature → | | 50 °C | 80 °C | 110 °C |
| Orientation ↓ | Number of contour lines ↓ | | | |
| | 0 | 360 ± 14 | 388 ± 26 | 442 ± 27 |
| 0° | 1 | 387 ± 3 | 415 ± 41 | 491 ± 59 |
| | 2 | 380 ± 13 | 415 ± 35 | 556 ± 80 |
| | 0 | 35 ± 5 | 135 ± 92 | 174 ± 168 |
| 45° | 1 | 435 ± 91 | 435 ± 84 | 360 ± 26 |
| | 2 | 572 ± 21 | 412 ± 27 | 524 ± 11 |
| | 0 | 80 ± 51 | 128 ± 101 | 198 ± 125 |
| 90° | 1 | 401 ± 54 | 482 ± 55 | 574 ± 32 |
| | 2 | 382 ± 41 | 571 ± 18 | 515 ± 48 |
